# Practical Notes on Diagnosis and Treatment

**Published:** 1907-05-04

**Authors:** 


### PRACTICAL NOTES ON DIAGNOSIS AND TREATMENT.

**Cystitis of Pregnancy.**

THE following is recommended: Camphor 1½ gr., opium ⅛ gr. Make one pill. Give five or six daily. If the cystitis is purulent, use boric acid injections, 2 per cent.

**Carbuncles.**

IN a spreading carbuncle the best way to stop it is by injections of carbolic acid—a pretty strong one is desirable—1 in 30. Two or three minims injected at short intervals round the carbuncle will speedily prevent it spreading.—*Dr. Radcliffe Crocker.*

**Stricture of the Urethra.**

GRADUAL dilatation should be applied in the first instance, I think, to almost every form of stricture, and it is only after the failure to make the tube sufficiently pervious by such means that one should resort to any more severe measures.—*Sir Wm. MacCormac.*

**Menorrhagia and Metrorrhagia.**

LET me name another drug from which I have seen useful results. I refer to oxide of silver. This may be given in the form of a pill, half a grain, one grain or two grains in each. I have known it check hæmorrhage where other drugs have failed.—*Sir Alex. Russell Simpson.*

**Œsophageal Stricture.**

IN passing an œsophageal bougie bear in mind that the entrance into the stomach is probably at least 13½ or 14 inches from the incisor teeth, and may be as much as 16 inches. I should not be sure, if I passed a tube for only 16 inches, that it had actually passed into the stomach.—*Mr. Butlin.*

**Intestinal Indigestion.**

A NUMBER of patients who complain of precordial pain and palpitation are the victims of imperfect intestinal digestion. Flatulent distention of the bowel, and especially of the transverse colon, will be found on examination. The following, administered in a cachet, often gives great relief:—Salol grs. 5, pancreatin grs. 2, oleo-resin of capsicum minim 1.

**Hæmorrhage after Tonsillotomy.**

MIX three parts of tannic acid and one part of gallic acid with a few drops of water and knead into a hard mass. From this take a piece sufficient to form a ball the size of a small marble. Rub this firmly against the bleeding surface, making counter pressure with the palm of the other hand placed on the side of the head over the region of the tonsil. I have never known this to fail.—*Mr. Mark Hovell.*

**Housemaid's Knee.**

YOU may simply tap the bursa with an exploring trocar—the smaller the trocar the better—and draw off the fluid. At least three or four "sittings" should be occupied in emptying the bursa. About a fourth of the fluid should be taken away on each occasion, and during the whole time firm pressure by means of a pad and bandage should be maintained. In this way a good and permanent result is more likely to be obtained than by emptying the cyst all at once.—*Sir W. H. Bennett.*

**Erythema Multiforme.**

A LONG course of arsenic is very useful in preventing the recurrence of attacks of erythema multiforme.—*Mr. Jonathan Hutchinson.*

**Ankle Clonus.**

ANKLE CLONUS may be obtained in hysteria, perhaps not so well marked as is often the case in organic disease, but yet sufficiently distinct to embarrass the diagnosis.

**Morphine in Aortic Disease.**

IN cardiac cases, and especially in aortic cases, when the patient is much distressed from insomnia, I have no reason to regret the use of hypodermics of morphine up to ⅙ of a grain.—*Dr. Francis Warner.*

**Migraine.**

DURING an attack of migraine the feet are often found to be very cold, and the adoption of measures to restore warmth improves the circulation, and often also gives at least some measure of relief from the headache.

**High Temperatures in Biliary Colic.**

IN many cases of biliary colic associated with jaundice there is present an ague-like temperature, which in many respects resembles an attack of urethral fever. There is a rigor, followed by a high temperature, which usually falls suddenly, the fall being often attended by free sweating.

**Locomotor Ataxia.**

THE two factors in the treatment of locomotor ataxia to which I should attach most importance are the use of rest, with counter-irritation of the spine, and the administration of chloride of aluminium. This drug is freely soluble in water, and may be given in doses of 2 to 4 grains.—*Dr. James Taylor.*

**Itching in Scarlet Fever.**

THIS is sometimes intolerable, and will keep a child restless and irritable. I find nothing more useful than sponging the body with a warm solution of sodium carbonate (grs. x. to ℥j.) to which a little mucilage has been added. It forms a soothing and grateful application to an inflamed skin.—*Dr. Seymour Taylor.*

**Diarrhœa in Enteric Fever.**

THIS is by no means always present. Indeed in many cases constipation is a marked feature of the disease. So long as the motions do not exceed four daily no attempt to check them should be made. When this number is exceeded opium may be given—preferably as a starch and opium enema, say, 20 minims of tincture of opium with 2 ounces of thin starch.

**Internal Hæmorrhoids**

DR. J. MCLEOD relates the cure of a case of internal hæmorrhoids which had troubled the patient, a man of 40 years, for more than 12 years. The cure is attributed to the use of the following suppositories: Chrysarobin gr. 1, iodoform gr. ¼, extract belladonna gr. ⅙, cocaine hydrochloride gr. ¼, cacao butter grs. xxx. One was used daily, and the patient was instructed to adopt the "squatting" position at stool.

